# Value of information analysis of an early intervention for subthreshold panic disorder: Healthcare versus societal perspective

**DOI:** 10.1371/journal.pone.0205876

**Published:** 2018-11-07

**Authors:** Robbin H. Ophuis, Joran Lokkerbol, Juanita A. Haagsma, Mickaël Hiligsmann, Silvia M. A. A. Evers, Suzanne Polinder

**Affiliations:** 1 Department of Public Health, Erasmus University Medical Center, Rotterdam, The Netherlands; 2 Centre of Economic Evaluation, Trimbos Institute (Netherlands Institute for Mental Health and Addiction), Utrecht, The Netherlands; 3 Department of Health Services Research, Care and Public Health Research Institute (CAPHRI), Maastricht University, Maastricht, The Netherlands; Ottawa Hospital Research Institute, CANADA

## Abstract

**Background:**

Panic disorder is associated with high productivity costs. These costs, which should be included in cost-effectiveness analyses (CEA) from a societal perspective, have a considerable impact on cost-effectiveness estimates. However, they are often omitted in published CEAs. It is therefore uncertain whether choosing a societal perspective changes priority setting in future research as compared to a healthcare perspective.

**Objectives:**

To identify research priorities regarding the cost-effectiveness of an early intervention for subthreshold panic disorder using value of information (VOI) analysis and to investigate to what extent priority setting depends on the perspective.

**Methods:**

We calculated the cost-effectiveness of an early intervention for panic disorder from a healthcare perspective and a societal perspective. We performed a VOI analysis, which estimates the expected value of eliminating the uncertainty surrounding cost-effectiveness estimates, for both perspectives.

**Results:**

From a healthcare perspective the early intervention was more effective at higher costs compared to usual care (€17,144 per QALY), whereas it was cost-saving from a societal perspective. Additional research to eliminate parameter uncertainty was valued at €129.7 million from a healthcare perspective and €29.5 million from a societal perspective. Additional research on the early intervention utility gain was most valuable from a healthcare perspective, whereas from a societal perspective additional research would generate little added value.

**Conclusions:**

Priority setting for future research differed substantially according to the perspective. Our study underlines that the health-economic perspective of CEAs on interventions for panic disorder must be chosen carefully in order to avoid inappropriate choices in research priorities.

## Introduction

The results of model-based cost-effectiveness analyses (CEAs) are surrounded by uncertainty as the available information about costs and effectiveness of interventions in healthcare is rarely perfect [1]. This uncertainty makes it difficult to guide policy makers because there is a risk of reimbursing suboptimal treatment options [2, 3]. The increasing financial pressure on healthcare systems emphasizes that decision uncertainty should be minimized.

Reimbursement decisions based on CEAs require a willingness-to-pay (WTP) threshold, which represents the maximum amount society is willing to pay for health gains. In cost-effectiveness research, health gains are preferably expressed in quality adjusted life-years (QALYs) as QALYs allow for comparison between health conditions due the generic character of this outcome measure. When a WTP threshold for a QALY is determined, the net monetary benefit (NMB) of the treatment alternatives of interest can be calculated by multiplying the WTP by the QALYs gained and subtracting the additional costs. The treatment alternative with the highest expected NMB is the preferred option.

A value of information (VOI) analysis estimates the expected value of eliminating the uncertainty surrounding cost-effectiveness estimates [1, 4]. This uncertainty can be minimized by performing additional research. Interventions might not be reimbursed because there is too much uncertainty about the right choice to be made. In that case, a VOI analysis can assist policy makers in deciding whether it is worthwhile to invest in additional research that could contribute to the reduction of this uncertainty. VOI also informs which specific model parameters might benefit the most from additional research [1, 2]. For example, this additional research can pertain to healthcare costs, productivity costs, or treatment effectiveness.

The costs outside the healthcare sector are substantial among patients with mental disorders [5–8]. In the Netherlands, these costs are higher than the healthcare costs for panic disorder as almost 90% of the societal costs is estimated to consist of productivity costs [9]. A CEA performed from a societal perspective includes both healthcare costs and costs outside the healthcare sector. According to guidelines CEAs should be performed from a societal perspective in most countries [10], including the Netherlands [11], but many of these studies on panic disorder are performed from a healthcare perspective [12]. Although one could expect that the cost-effectiveness of interventions for panic disorder would improve when including costs outside healthcare, only few studies support this assumption. More important, the role of the societal perspective in setting future research priorities remains uncertain. Because CEAs from a societal perspective include additional parameters on costs outside healthcare, the research priorities identified by the VOI from a societal perspective might differ from the research priorities as identified by the analysis from a healthcare perspective.

In a previously published paper [13], we constructed a health economic model to assess the cost-effectiveness of adding a cognitive behavioral therapy (CBT) based early intervention for adults with subthreshold panic disorder (STHPD) to the existing health care (usual care) for people with panic disorder in the Netherlands. We adopted a societal perspective as the model included both healthcare costs and productivity costs. When the costs outside healthcare are less relevant for a decision maker, a VOI analysis applied to a CEA from a societal perspective may identify inappropriate research priorities. This applies to panic disorder in particular because the productivity costs have a substantial impact on cost-effectiveness estimates [13].

To our knowledge, a VOI analysis applied to CEAs of interventions for (subthreshold) panic disorder has not yet been published. It therefore remains unclear whether additional research into specific model parameters to reduce uncertainty has additional value, and it also remains unclear whether these research priorities depend on the perspective that is applied. This information is valuable because decision makers not only have to decide which treatment alternative to adopt, but also whether more research regarding the decision is desirable [4]. Moreover, the new Dutch guidelines for health economic evaluations require quantification of uncertainty by means of VOI analysis [11]. In this study, we therefore aim to identify research priorities regarding the cost-effectiveness of a CBT-based early intervention for STHPD using VOI analysis and to investigate to what extent priority setting depends on the perspective.

## Methods

### Cost-effectiveness model

The VOI analysis was applied to a previously developed model-based CEA. Based on this Markov model constructed in Microsoft Excel [13], we analyzed the cost-effectiveness of adding a CBT-based early intervention for adults with STHPD to the existing health care for people with panic disorder in the Netherlands. In brief, the modelled population was classified into three health states based on the presence and severity of panic symptoms: panic free, STHPD, and panic disorder. Death was added as an absorbing health state. Patients with STHPD experience clinically relevant panic symptoms, but they do not meet the established diagnostic criteria for panic disorder [14]. In the Netherlands, the prevalence of STHPD is comparable to the prevalence of panic disorder (1.9% versus 2.2%) [15].

We applied a cycle length of one year, meaning that patients remained in the same health state or switched to a connecting health state after one year. The time horizon of the analysis was five years. Because the long-term effect of the CBT-based early intervention is unknown we decided that applying the six months treatment effect to a CEA with a time horizon of >5 years would be inappropriate. In the previous cost-effectiveness study [13], a scenario analysis with a ten-year time horizon was tested. The results were comparable to the results based on a time horizon of five years. QALYs and costs of the intervention and usual care scenarios were the outcome of the model. We expressed monetary outcomes in euros (€) for the 2018 price level. More information on the model and its structure and underlying assumptions are published elsewhere [13]. We assigned distributions to the model parameters in order to include the uncertainty surrounding them. In line with Briggs (2005) [16], cost parameters were assigned a gamma distribution, and utility parameters were assigned a beta distribution. A simulation of 10,000 model iterations was performed by drawing random values for the input parameters, which were used to estimate the mean incremental cost-effectiveness. The model parameters, 95% confidence intervals, probabilistic distributions, and sources are reported in the supporting information (S1 Table). The previously developed model was used as a basis for the current analyses [13], but several aspects were adjusted. Costs were uprated to a more recent price level (2018), utility data were assigned a beta distribution, and the early intervention relative risk that was multiplied with the STHPD to panic disorder transition probability was included in the probabilistic sensitivity analysis (log-normal distribution).

### Treatment options

Usual care consists of the currently available care for panic disorder in the Netherlands covering psychotherapy, pharmacotherapy, or a combination of these therapies [13]. We investigated the effect of adding a CBT-based early intervention for STHPD to the usual care for panic disorder in the Netherlands. The early intervention called ‘Don’t Panic’ is a group-based course for 6–12 adults covering CBT aspects such cognitive restructuring and in vivo exposure [17, 18]. The intervention consists of eight weekly sessions guided by a prevention worker and mental health clinician. More detailed information about the treatment alternatives, adherence rates, and panic disorder intervention coverage rates is published elsewhere [13].

### Cost-effectiveness analysis

In addition to the previously published CEA from a societal perspective [13], we also calculated the cost-effectiveness from a healthcare perspective by excluding the productivity costs. The societal perspective often refers to the addition of merely productivity costs, while other non-healthcare costs could be relevant as well [19]. In this research paper, however, the term societal perspective refers to the inclusion of healthcare costs and productivity costs. Therefore, the difference between the analyses from both perspectives solely consists of the inclusion of productivity costs. Mean costs and QALYs were estimated for usual care and the CBT-based early intervention. We applied annual discount rates for both costs (4%) and effects (1.5%) as recommended by the Dutch health economic guidelines [13, 20]. The incremental cost-effectiveness ratio (ICER) was calculated by dividing the difference in healthcare and societal costs between both scenarios by the difference in QALYs. We constructed a cost-effectiveness acceptability curve, which shows the cost-effectiveness probability of the usual care and early intervention scenarios for a range of WTP threshold values. The early intervention was deemed cost-effective when the ICER was lower than the WTP of €20,000 per QALY. The WTP threshold of €20,000 per QALY is a recommended threshold in the Netherlands for diseases with a relatively low disability weight [21].

### VOI analysis

In the VOI analysis, the parameter uncertainty in the model was valued. Our VOI analyses consist of two parts: the expected value of perfect information (EVPI), and the expected value of partial perfect information (EVPPI). The EVPI represents the estimated value of eliminating the uncertainty in the model. More specific, the EVPI places an upper boundary on the costs of performing additional research to eliminate the uncertainty. It can thus be interpreted as the maximum of costs society should be willing to pay for additional evidence that eliminates the current decision uncertainty. Having perfect information regarding the cost-effectiveness of adding a CBT-based early intervention to usual care for panic disorder would protect against making suboptimal decisions regarding reimbursement and implementation as the cost-effectiveness estimate based on the model would be certain. The EVPI informs policy makers whether it is worthwhile to invest in further research that will contribute to the reduction of the current decision uncertainty.

In the first step, the NMB for every model iteration of each treatment alternative (the additional CBT-based early intervention and usual care) was calculated by multiplying the WTP by the QALYs and subtracting the costs. Thereafter we identified the preferred treatment alternative, which is the alternative that maximizes the NMB for the given WTP threshold based on current information in the model. Thereafter, the NMB when having perfect information (the EVPI) was calculated. Within each simulation run, the individual EVPI was equal to the difference between the NMB of the optimal treatment alternative based on the current information and the potential maximum NMB. The individual EVPI thus equals zero when the recommended option by the model under current uncertainty has the highest NMB. The individual EVPI was averaged over all 10,000 model runs. This method is illustrated in Table 1 for the analysis from a healthcare perspective. The same methods were applied to the analyses from a societal perspective.

**Table 1:** Calculating the individual expected value of perfect information (EVPI), example for the analysis from a healthcare perspective.

	NMB^a^			
Model runs	CBT-based early intervention	Usual care	Maximum NMB	Net benefit gained when having perfect information
1	63,193	65,163	65,163	65,163–63,193 = 1,970
2	56,206	56,904	56,904	56,904–56,206 = 698
3	62,997	61,630	62,997	62,997–62,997 = 0
4	63,466	62,504	63,466	63,466–63,466 = 0
5	62,230	61,703	62,230	62,230–62,230 = 0
…	…	…	…	…
10,000	59,159	59,770	59,770	59,770–59,159 = 611
**Mean**^**b**^	**60,831**	**60,643**	**61,483**	**61,483–60,831 = 652**^**c**^

EVPI: expected value of perfect information, NMB: net monetary benefit.

^a^ NMB was calculated by multiplying QALYs by the WTP threshold of €20,000 per QALY and subtracting costs. The early intervention resulted in the highest overall NMB for both perspectives.

^b^ The mean of all 10,000 model iterations.

^c^ The individual EVPI (€652) was calculated as the difference between the expected NMB with perfect information and the expected NMB with current information.

The population EVPI was calculated from a healthcare and a societal perspective by multiplying the average individual EVPI values by the number of STHPD patients in the Netherlands who receive the early intervention in the model (n = 198,896). The population EVPI values from a healthcare perspective and a societal perspective were plotted against a range of different WTP thresholds, resulting in a population EVPI curve.

Although the population EVPI provides insight into the NMB gained when having perfect information, it remains unclear which parameters in the model are associated with the greatest uncertainty. If the uncertainty in the current analysis mainly results from the healthcare costs parameters, it might not be worthwhile to further investigate the parameters on treatment effectiveness.

To identify for which parameters future research is worthwhile for both perspectives, we calculated the EVPPI. The EVPPI is calculated as the difference between the expected NMB of a decision made with perfect information on a group of parameters and the expected NMB with current information on that group of parameters. In order to calculate the EVPPI, we performed the model simulations (inner loop) for a fixed randomly drawn value for the parameter(s) of interest given a WTP threshold of €20,000 per QALY. Subsequently, new fixed values of the same parameters were drawn and the simulation was performed again (outer loop). In line with Mohseninejad et al. [6], the number of inner loops was set at 1,000 and the number of outer loops at 100. The simulations were repeated to check for stability of the results. We calculated the EVPPI for the following model parameters or groups of parameters: intervention costs, health-state utilities, utility gain after receiving the early intervention, healthcare costs, the early intervention relative risk that was multiplied with the STHPD to panic disorder transition probability, and the productivity costs (societal perspective only).

## Results

### Cost-effectiveness

**Fig 1:**
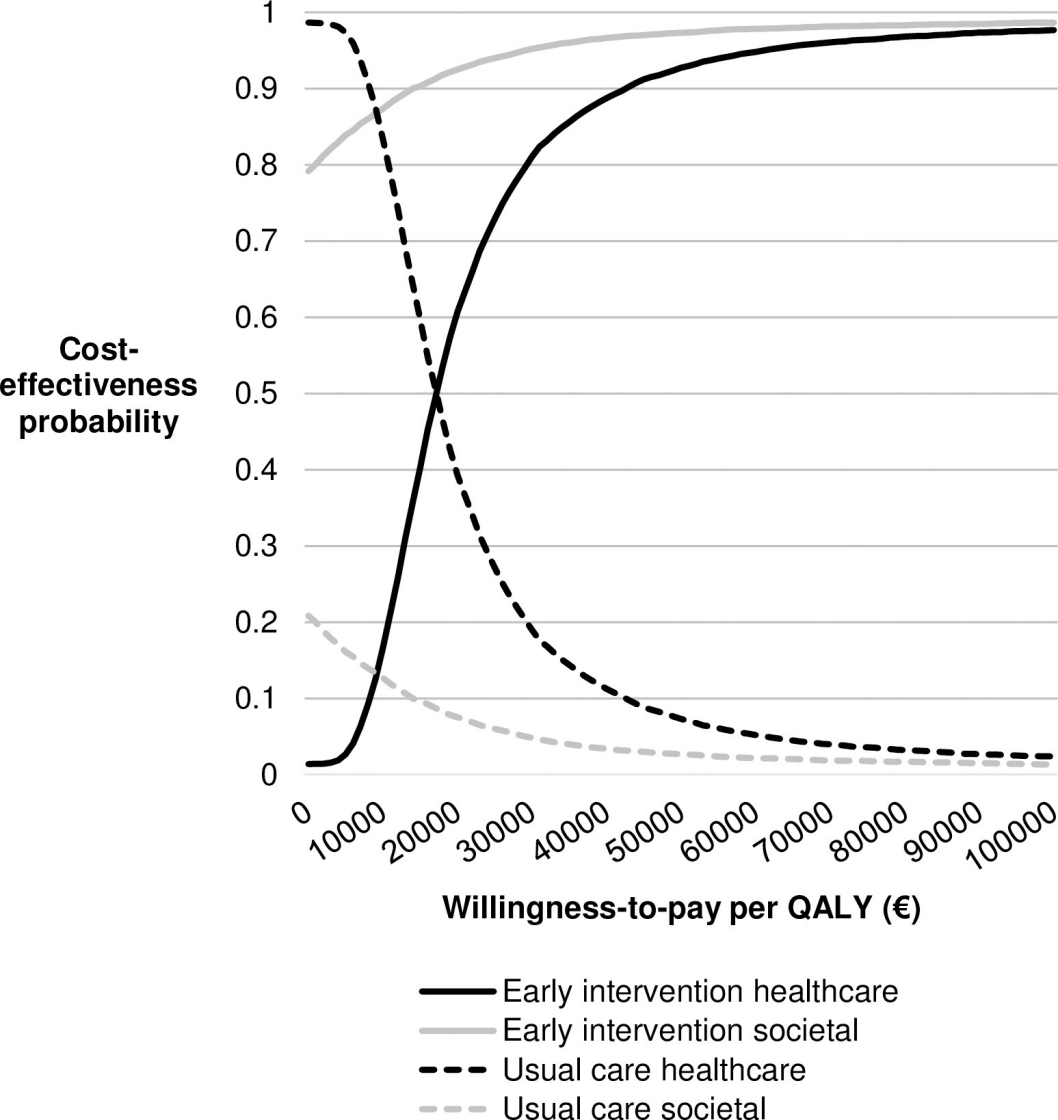
Cost-effectiveness acceptability curve of the additional CBT-based early intervention and usual care.

Estimates of the QALYs and costs per treatment alternative are shown in Table 2. S1 Fig shows a scatterplot including the incremental QALYs and costs for the analyses from both perspectives resulting from the 10,000 model iterations. From a healthcare perspective, the ICER of the early intervention versus usual care resulted in €17,144 per QALY gained. The results from societal perspective showed that the intervention was cost-saving, with an ICER of €-16,023 per QALY gained. Fig 1 shows the cost-effectiveness acceptability curves, which shows the cost-effectiveness probability of both treatment options for a range of different WTP thresholds. Given a WTP threshold of €20,000 per QALY, the estimated cost-effectiveness probability for the early intervention was 61% from a healthcare perspective and 93% from a societal perspective.

**Table 2:** Estimates of discounted costs and QALYs per patient and incremental costs and QALYs.

	Mean QALYs (95% CI)	Mean healthcare costs (95% CI)	Mean productivity costs (95% CI)
Additional CBT-based early intervention	3.44 (3.19–3.68)	€8,034 (4,941–11,952)	€50,912 (35,143–70,055)
Usual care	3.28 (3.08–3.47)	€4,934 (2,904–7,481)	€58,004 (42,469–75,931)
Incremental	0.16 (0.02–0.32)	€3,100 (1,002–5,871)	-€7,092 (-18,469–2,997)

CBT: cognitive behavioral therapy, CI: confidence interval, QALYs: quality adjusted life-years

### VOI analysis

As an example, Table 1 presents the generated NMBs for both treatment options for six model runs of the CEA from a healthcare perspective. The early intervention had the highest NMB value for the CEAs from both perspectives, and therefore the early intervention was the preferred treatment option. Therefore, no benefits were gained for model runs that identified the early intervention as the treatment option with the highest NMB. The individual EVPI values were €652 from a healthcare perspective and €148 from a societal perspective. Multiplying these values with the target population (n = 198,896) resulted in population EVPIs of €129.7 million from a healthcare perspective and €29.5 million from a societal perspective given a WTP threshold of €20,000 per QALY. Reducing all parameter uncertainty was thus valued at a total of €129.7 and €29.5 million, respectively.

**Fig 2:**
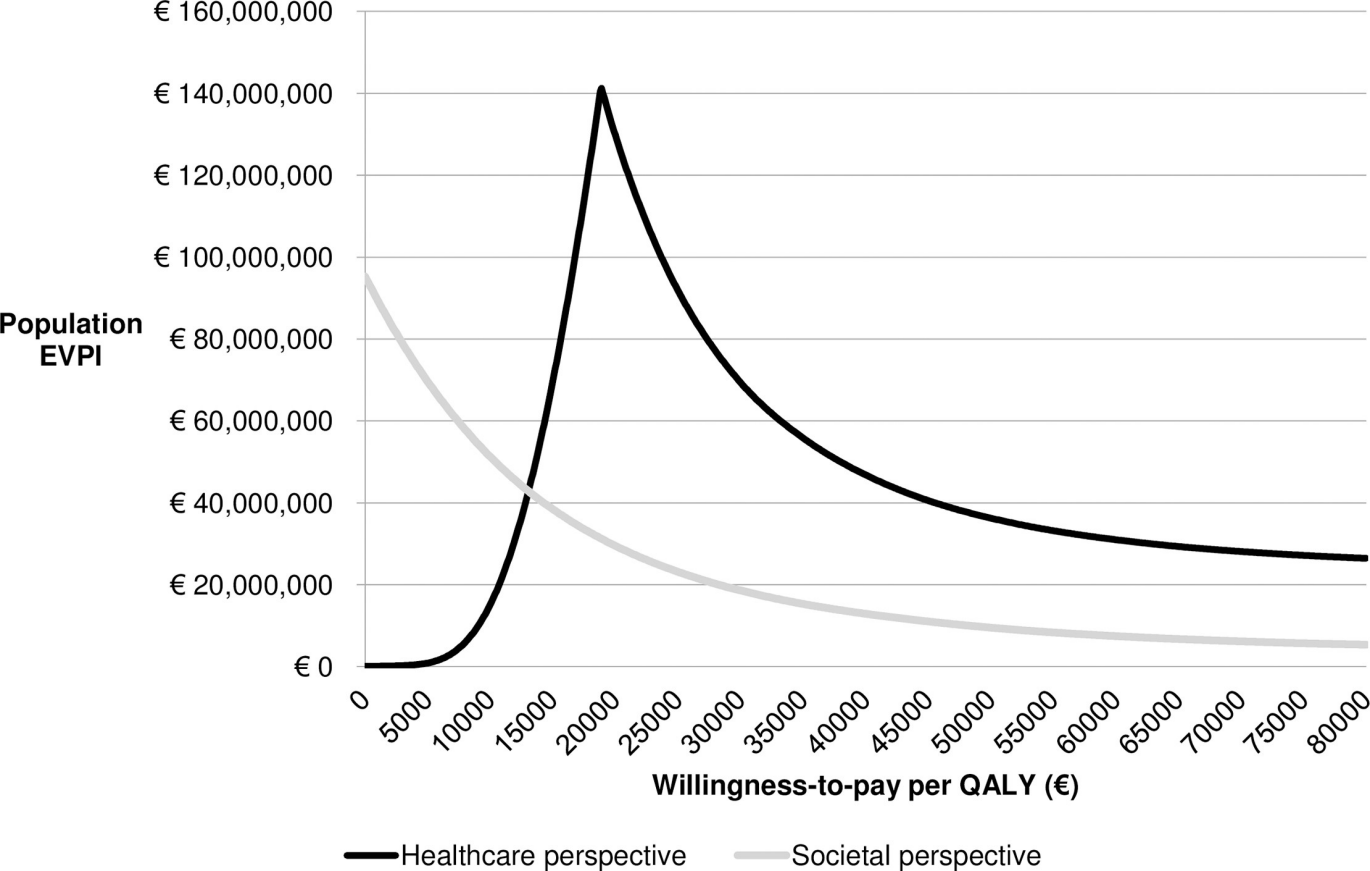
The population expected value of perfect information (EVPI) for different willingness-to-pay thresholds.

Fig 2 presents the estimated population EVPI values from both healthcare and societal perspectives over a range of WTP values. The population EVPI from a healthcare perspective showed a maximum of €141.2 million at a WTP of €18,900 per QALY. At this threshold, decision uncertainty was the highest. The probability that the additional early intervention was cost-effective increased for larger WTP thresholds, and therefore the EVPI decreased. Because the early intervention was expected to be more effective at lower costs from a societal perspective, the cost-effectiveness probability further increased for larger WTP thresholds. As decision certainty increased with WTP, the societal EVPI declined.

**Fig 3:**
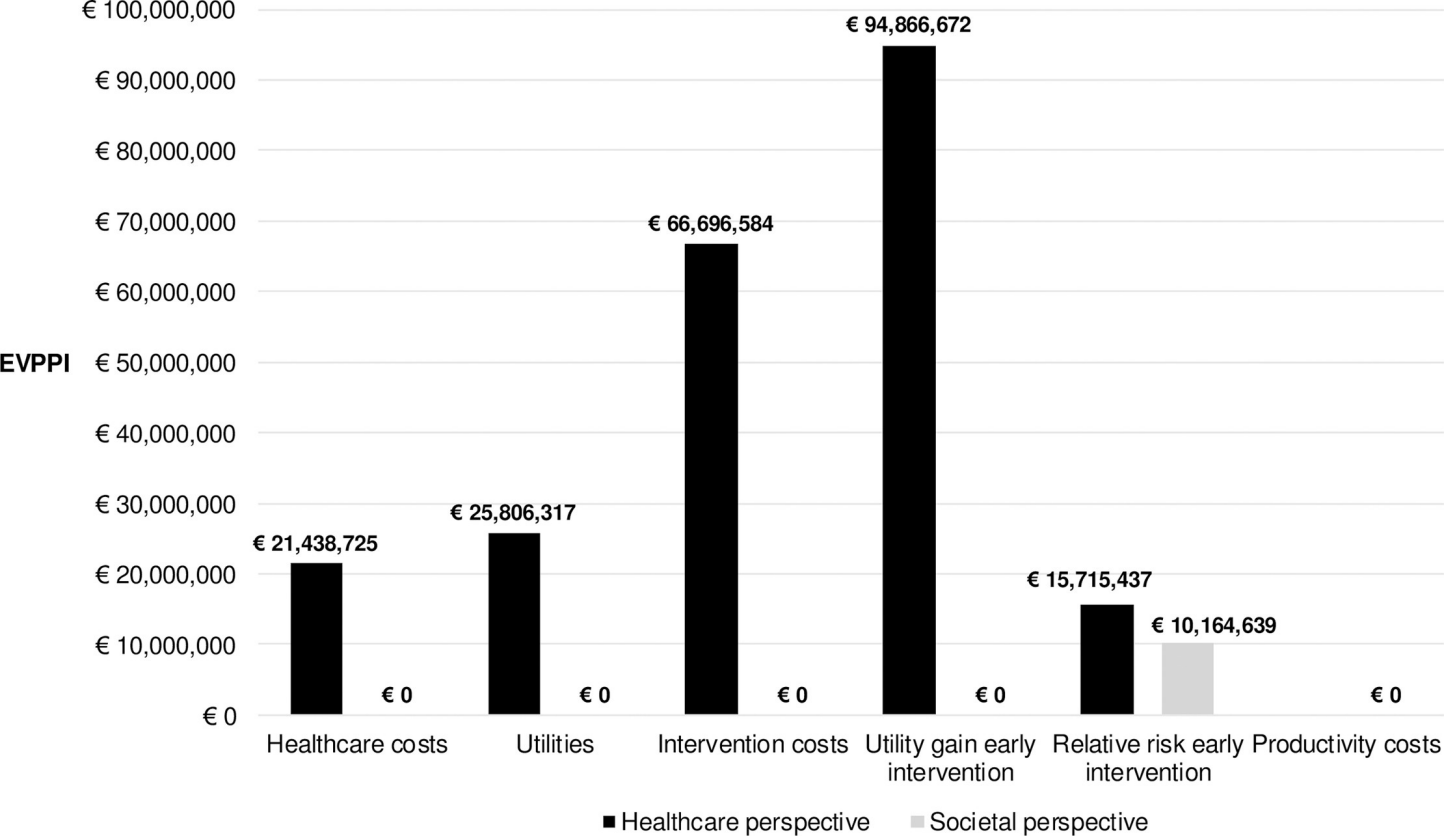
Expected value of partial perfect information (EVPPI) from both perspectives given a willingness-to-pay threshold of €20,000 per QALY.

The uncertainty of subsets of parameters was valued in the EVPPI given a WTP threshold of €20,000 per QALY (Fig 3). Because the model parameters interact within the model structure and because not all parameters are considered, the EVPPIs do not sum up to the overall EVPIs for each perspective [22, 23]. When the CEA is performed from a healthcare perspective, the early intervention utility gain parameter contributed to most of the uncertainty (73% of total EVPI), followed by the intervention cost parameters and the health-state utility parameters (51% and 20% of total EVPI). The population EVPPIs for these groups of parameters equaled €94.9 million, €66.7 million, and €25.8 million, respectively. The EVPPIs for the healthcare cost parameters and the relative risk for the early intervention equaled €21.4 and €15.7 million. When the cost-effectiveness analysis was performed from a societal perspective, the EVPPI of the early intervention relative risk parameter equaled €10.2 million. The EVPPIs for the remaining (groups of) parameters were equal to zero, including the productivity costs, which indicates that there was no expected value of further research into these parameters.

## Discussion

In this study, we aimed to identify research priorities regarding the cost-effectiveness of a CBT-based early intervention for STHPD using VOI analysis and to investigate to what extent priority setting depends on the perspective. The decision uncertainty was relatively high when a healthcare perspective was applied, and eliminating uncertainty was valued at a total of €129.7 million for a WTP of €20,000 per QALY. The decision uncertainty in our model is comparable to other cost-effectiveness analyses on psychological interventions in mental healthcare that applied a healthcare perspective [6, 24]. Additional research on the early intervention utility gain in the model was most valuable from a healthcare perspective. However, when a societal perspective was applied, the decision uncertainty was minimal and additional research was expected to have little added value. The EVPI for specific (groups of) parameters (EVPPI) equaled zero from a societal perspective, except for the early intervention relative risk parameters. These results indicate that additional research on the healthcare costs, productivity costs, health-state utilities, early intervention utility gain, and intervention costs considered in the model would not generate value from a societal perspective.

Our results indicate that the cost-effectiveness estimates and the potential value of additional research substantially differed when we applied a societal perspective instead of a healthcare perspective. From a societal perspective, additional research would generate little additional value whereas further research from a healthcare perspective could be considered worthwhile, especially for the utility gain associated with the early intervention. When healthcare policy makers decide to invest in a CBT-based early intervention for STHPD at a WTP of €20,000 per QALY, the cost-effectiveness probability that the early intervention is cost-effective is only 61%. Because this decision uncertainty is relatively high, reducing decision uncertainty would be beneficial for healthcare policy makers. Investing in additional research on the early intervention utility gain, intervention costs, and health-state utilities in order to reduce uncertainty could be considered. For employers or others investors for whom the societal perspective is relevant, on the other hand, additional research is not likely to be worthwhile as additional information within the probabilistic sensitivity analysis would not generate additional value. It is unlikely that investing in additional research on the parameters included in the VOI will yield extra information regarding the decision from a societal perspective, which emphasizes the importance of identifying the relevant perspective for the CEA.

In a comparable VOI analysis on a minimal intervention for depression [6], however, additional research on the costs outside healthcare generated most value from a societal perspective. Additional research on this model parameter was considered worthwhile. The same WTP threshold of €20,000 per QALY was applied [6]. A possible explanation for this difference could be that in our study the intervention was more cost-saving. Consequently, the uncertainty surrounding the non-healthcare costs parameters might have been less influential as even for lower thresholds the early intervention was cost-saving. Another VOI analysis in the field of mental health was applied to an intervention on reducing juvenile delinquency [3]. In that study, the healthcare costs and intervention costs were relatively uncertain, which is comparable to our findings from a healthcare perspective. However, comparing results between different CEAs and VOI analyses is difficult because of the different model structures, input parameters, and parameters included in the probabilistic sensitivity analysis and EVPPI.

To our knowledge, our study is the first to apply a VOI analysis to cost-effectiveness research in the field of (subthreshold) panic disorder. A strength of the application of this analysis is that it values the uncertainty surrounding the cost-effectiveness estimate, which provides valuable input for policy decisions regarding investments in future research. There are also some limitations that require discussion. The VOI analysis in this article estimates the value of eliminating the parameter uncertainty of the presented model, but two other relevant sources of uncertainty may have influenced the results which were not captured by VOI analysis: structural and methodological uncertainty [25, 26].

Structural uncertainty is likely to be present in our model. For example, we did not apply different health-state transition rates for individuals who received any treatment for panic disorder due to a lack of available information [13]. Instead, the effectiveness of interventions was reflected in utility gains. It was assumed that patients who received interventions for panic disorder had the same chance of remission as patients who did not receive an intervention [13]. Therefore, the effect of the interventions for panic disorder might have been underestimated. We did not calculate the EVPPI for the health state transition parameters since they were fixed. We consider this as a limitation, because in a comparable VOI study the health-state transition rates were the most uncertain [3].

The second source of uncertainty is methodological uncertainty, which relates to the analytical methods used in the model. One aspect related to this uncertainty is the WTP threshold, which requires attention given its importance for our study. We applied a WTP of €20,000 per QALY in our study, which we used for the CEAs and VOI of both health economic perspectives. However, a WTP of €20,000 is mostly used for a healthcare setting [6, 27]. Although the application of different WTP thresholds for CEAs from a healthcare and a societal perspective is uncommon in published CEAs, it has been mentioned that using a different WTP according to perspective might be more appropriate [28, 29]. A reason is that from a societal perspective threshold values should reflect the consumption of health from a societal perspective, while from a healthcare perspective WTP thresholds would reflect the marginal value of health provided for by a publicly financed health care system, which are not always the same. As it is likely that a societal WTP would be larger than the healthcare WTP and because the addition of productivity costs in a CEA improves cost-effectiveness in most cases we can expect that the cost-effectiveness would always be more favorable from a societal perspective when an intervention is more effective than the comparator. We could therefore expect that the value of additional research as identified by VOI from a societal perspective would decrease as the intervention already has less uncertainty about the most cost-effective option compared to the healthcare perspective.

The effect of the early intervention that was applied in the model was largely based on one trial (n = 216) [18]. Although our results indicate that from a societal perspective the value of additional research would be minimal compared to the healthcare perspective, it remains uncertain to what extent the results of this trial can be extrapolated to the general population in the Netherlands. Therefore, additional research investigating the effect of the early intervention is still desirable. VOI is limited to the uncertainty surrounding parameter estimates, but neither the quality nor the interpretation of the parameters is considered. These types of uncertainty are not reflected in the EVP(P)I. Assuming that the choices in our model are a good approximation of reality, additional research is expected to have little additional value from a societal perspective.

In order to incorporate the treatment effect of the early intervention in the transition probabilities, we applied a log-normal distributed relative risk to the STHPD to panic disorder transition. A limitation of our study is that the remaining transition parameters in the model were fixed. However, as the intervention’s cost-effectiveness is primarily the result of the intervention’s reduction in the STHPD to panic disorder transition, the uncertainty in the other transition rates can be considered of second-order importance.

Although VOI analysis quantifies the value of uncertainty, in general and for specific model parameters, its practical application remains difficult [30]. The EVPI is estimated on the assumption that only cost-effectiveness outcomes guide decision making. However, additional decision makers’ constraints such as the maximum budget impact and health equity are usually not taken into account [30]. For future research and policy decisions on early interventions for panic disorder, it is therefore important that such considerations are identified.

In conclusion, our results showed that research priorities regarding an early intervention for STHPD differed according to the health-economic perspective applied to the CEA. The VOI analysis indicated that additional research to eliminate uncertainty surrounding the cost-effectiveness estimate is worthwhile when the CEA is performed from a healthcare perspective, but less so when a societal perspective was applied. When the probability that an intervention is more effective as well as cost-saving is high, the value of additional research is likely to be minimal. Because performing a VOI analysis is recommended in the new Dutch guidelines for economic evaluations in healthcare [11], a conscious consideration of the health-economic perspective used for CEAs on interventions for panic disorder is therefore important. Our study underlines that the health-economic perspective of CEAs on interventions for panic disorder must be chosen carefully in order to avoid inappropriate choices in research priorities.

## Supporting information

S1 Table Model input. (PDF)

S1 Fig Scatterplot incremental costs-effectiveness. (PDF)
