# Supplementary material for: Value of information analysis of an early intervention for subthreshold panic disorder: Healthcare versus societal perspective
Source: PLoS One. 2018 Nov 7;13(11):e0205876. doi: 10.1371/journal.pone.0205876 (PMC6221282; doi:10.1371/journal.pone.0205876)
Supplement: S1 Table — (PDF) [file pone.0205876.s001.pdf]

S1 Table

S1 Table: Model parameters

	Value (95% CI)	Probabilistic distribution	Source
**Discount rates**			
Costs	4%	Fixed	Dutch Guideline for Health Economic Evaluations [1]
Outcomes	1.5%	Fixed	Dutch Guideline for Health Economic Evaluations [1]
**Transition probabilities**			
PF to STHPD	0.0016	Fixed	Calculations based on epidemiology [2, 3]
STHPD to PF	0.0815	Fixed	Calculations based on epidemiology [2, 3]
STHPD to PD	0.6037	Fixed	Calculations based on epidemiology [2, 3]
Relative risk early intervention[a]	0.524 (0.241-1.141)	Log-normal	Smit et al. [4]
PD to STHPD	0.5214	Fixed	Calculations based on epidemiology [2, 3]
Any state to death	0.0028	Fixed	Based on Dutch national statistics, averaged for the population aged 18-65 year [5]
**Annual healthcare costs**			
PF	€358 (158-559)	Gamma	Based on data by Batelaan et al. [2]
STHPD	€1,586 (134-3,037)	Gamma	Based on data by Batelaan et al. [2]
PD	€702 (309-1,095)	Gamma	Based on data by Batelaan et al. [2]
**Annual productivity costs**			
PF	€7,008 (3,889-10,126)	Gamma	Based on data by Batelaan et al. [2]
STHPD	€10,275 (5,019-15,531)	Gamma	Based on data by Batelaan et al. [2]
PD	€17,691 (9,818-25,564)	Gamma	Based on data by Batelaan et al. [2]
**Intervention costs**			
CBT[b]	€1,176 (526-2,094)	Gamma	[1], expert opinion
SSRI[c]	€1,128 (503-1,956)	Gamma	[1], expert opinion
TCA[d]	€1,200 (549-2,120)	Gamma	[1], expert opinion
Combination therapy[e]	€2,315 (1,219-3,735)	Gamma	[1], expert opinion

Early intervention	€955 (406-1,692)	Gamma	Smit et al. [4]
**Health state utilities**			
PF	0.869 (0.858-0.880)	Beta	Versteegh et al. [6]
STHPD	0.730 (0.665-0.794)	Beta	Based on Versteegh et al. [6] and Stouthard et al. [7]
PD	0.660 (0.608-0.713)	Beta	Based on Versteegh et al. [6] and Kaltenthaler et al. [8]
**Utility gain after intervention[f]**			
CBT	0.033	Normal	Based on Sanderson et al. [9] and Bandelow et al. [10]
SSRI	0.045	Normal	Based on Sanderson et al. [9] and Bandelow et al. [10]
TCA	0.045	Normal	Based on Sanderson et al. [9] and Bandelow et al. [10]
Combination therapy	0.057	Normal	Based on Sanderson et al. [9] and Bandelow et al. [10]
Early intervention	0.066	Normal	Based on Sanderson et al. [9] and Meulenbeek et al. [11]

CBT: cognitive behavioral therapy, PD: panic disorder, PF: panic-free, SSRI: selective serotonin inhibitor, STHPD: subthreshold panic disorder, TCA: tricyclic antidepressant.
[a] Applied to the STHPD to PD transition for the patients receiving the early intervention.
[b] 12 sessions in Basic mental health care.
[c] Defined Daily Dose for one year (average SSRI costs) plus 9.5 sessions (min. 8 and max.12) in specialized mental health care.
[d] Defined Daily Dose for one year (average TCA costs) plus 9.5 sessions in specialized mental health care.
[e] 12 general mental health institution sessions, Defined Daily Dose for one year (average TCA/SSRI costs) plus 9.5 sessions in specialized mental health care.
[f] Utility gain was based on the treatment effect size and utility conversion rates as described in the 'Raw model input utility gain' table on page 3. Effect sizes were normally distributed and conversion rates were beta distributed.
Note: costs are expressed in euros for the reference year 2018.

Raw model input utility gain

Parameter	Value (mean)	SE	Source
Effect size CBT	1.142	0.075	Bandelow et al. [10]
Effect size SSRI	1.651	0.135	Bandelow et al. [10]
Effect size TCA	1.729	0.185	Bandelow et al. [10]
Effect size pill placebo	1.124	0.115	Bandelow et al. [10]
Effect size psychological placebo	0.937	0.31	Bandelow et al. [10]
Effect size pharmacotherapy+CBT	1.587	0.19	Bandelow et al. [10]
Effect size early intervention	0.791	0.17	Based on Meulenbeek et al. [11]
**Multiplication factor for estimating utility gain (Effect size*factor)**			
Rating scale method	0.176	0.009	Sanderson et al. [9]
Time trade-off	0.109	0.011	Sanderson et al. [9]

CBT: cognitive behavioral therapy, SE: standard error, SSRI: selective serotonin inhibitor, TCA: tricyclic antidepressant.

References

1. Hakkaart-van Roijen L, Tan SS, Bouwmans CAM. Handleiding voor kostenonderzoek: Methoden en standaard kostprijzen voor economische evaluaties in de gezondheidszorg: College voor zorgverzekeringen; 2010.
2. Batelaan N, Smit F, de Graaf R, van Balkom A, Vollebergh W, Beekman A. Economic costs of full-blown and subthreshold panic disorder. J Affect Disord. 2007;104(1-3):127-36. PubMed PMID: 17466380.
3. Batelaan NM, de Graaf R, Penninx BW, van Balkom AJ, Vollebergh WA, Beekman AT. The 2-year prognosis of panic episodes in the general population. Psychol Med. 2010;40(1):147-57. PubMed PMID: 19400975.
4. Smit F, Willemse G, Meulenbeek P, Koopmanschap M, van Balkom A, Spinhoven P, et al. Preventing panic disorder: cost-effectiveness analysis alongside a pragmatic randomised trial. Cost Eff Resour Alloc. 2009;7:8. PubMed PMID: 19393084.
5. CBS Statline. Overledenen; belangrijke doodsoorzaken (korte lijst), leeftijd, geslacht 2016 [cited 2017 Feb 17]. Available from: http://statline.cbs.nl/Statweb/publication/?DM=SLNL&PA=7052_95&D1=0,92&D2=a&D3=0,5-15&D4=l&HDR=G1,G3&STB=T,G2&VW=T.
6. Versteegh M, Vermeulen K, Evers S, de Wit A, Prenger R, Stolk E. Dutch Tariff for the Five-Level Version of EQ-5D. Value in Health. 2016;19(4):343-52. doi: http://dx.doi.org/10.1016/j.jval.2016.01.003.
7. Stouthard MEA, Essink-Bot ML, Bonsel GJ, Barendregt JJM, Kramers PGN, Water H, et al. Disability weights for diseases in the Netherlands. 1997.
8. Kaltenthaler E, Brazier J, De Nigris E, Tumur I, Ferriter M, Beverley C, et al. Computerised cognitive behaviour therapy for depression and anxiety update: a systematic review and economic evaluation. Health Technol Assess. 2006;10(33):iii, xi-xiv, 1-168. PubMed PMID: 16959169.
9. Sanderson K, Andrews G, Corry J, Lapsley H. Using the effect size to model change in preference values from descriptive health status. Qual Life Res. 2004;13(7):1255-64. PubMed PMID: 15473504.
10. Bandelow B, Reitt M, Rover C, Michaelis S, Gorlich Y, Wedekind D. Efficacy of treatments for anxiety disorders: a meta-analysis. Int Clin Psychopharmacol. 2015;30(4):183-92. PubMed PMID: 25932596.
11. Meulenbeek P, Willemse G, Smit F, van Balkom A, Spinhoven P, Cuijpers P. Early intervention in panic: pragmatic randomised controlled trial. Br J Psychiatry. 2010;196(4):326-31. PubMed PMID: 20357312.
