# Supplementary figures and images for: Value of information analysis of an early intervention for subthreshold panic disorder: Healthcare versus societal perspective
Source: PLoS One. 2018 Nov 7;13(11):e0205876. doi: 10.1371/journal.pone.0205876 (PMC6221282; doi:10.1371/journal.pone.0205876)

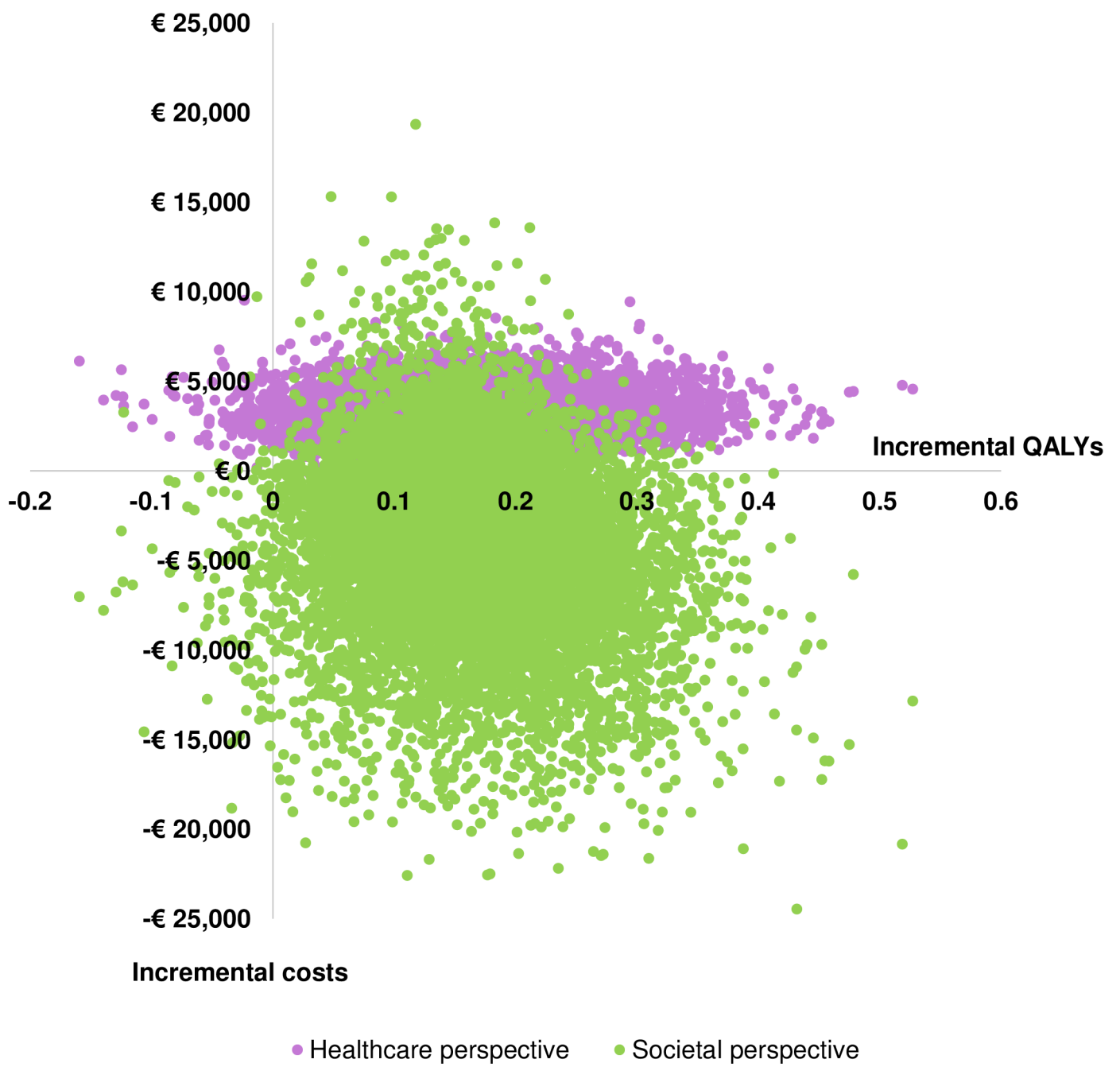

Supplement: S1 Fig — (PDF) [file pone.0205876.s002.pdf]
